# Nasal position of nasotracheal tubes: a retrospective analysis of intraoperatively generated three-dimensional X-rays during maxillofacial surgery

**DOI:** 10.1186/s40001-014-0055-7

**Published:** 2014-10-17

**Authors:** Lili Plümer, Gerhard Schön, Jan Klatt, Henning Hanken, Rainer Schmelzle, Philipp Pohlenz

**Affiliations:** Center for Anesthesiology and Intensive Care Medicine, Department of Anaesthesiology, University Medical Center Hamburg-Eppendorf, Hamburg, Germany; Center for Experimental Medicine, Department of Medical Biometry and Epidemiology, University Medical Center Hamburg-Eppendorf, Hamburg, Germany; Own medical practice of Oral and Maxillofacial Surgery, Hamburg, Germany; Center for Clinical Neurosciences, Department of Oral and Maxillofacial Surgery, University Medical Center Hamburg-Eppendorf, Hamburg, Germany; Own medical practice of Oral and Maxillofacial Surgery and of Plastic and Reconstructive Surgery, Hamburg, Germany; Department of Oral and Maxillofacial Surgery and of Plastic and Reconstructive Surgery, Red Cross Hospital, Bremen, Germany

**Keywords:** Intubation, Nasotracheal tube, Maxillary sinusitis

## Abstract

**Background:**

The aim of this retrospective investigation was to evaluate the position of the nasotracheal tube in the nose and to show its anatomical relationship with the maxillary sinus ostium.

**Methods:**

Fifty data sets from patients who had undergone endonasal intubation were analyzed for tube positioning. There was a drop-out of eight data sets due to missing information concerning tube size and mode. Tube positioning was determined at the maxillary sinus ostium in the intraoperatively generated three-dimensional X-ray data sets. The type of tube, the tube size, and the presence of maxillary sinusitis were analyzed 30 minutes after intubation.

**Results:**

The tube was positioned in the middle nasal meatus in 35 (83.3%) patients and not in the middle nasal meatus in 7 (16.7%) patients. The difference in comparison with equal distribution was significant (*P* <0.001). The test value was 83.3; the 95% confidence interval of the test value was 68.6 to 93.0%. Maxillary sinusitis was diagnosed as a chance finding in 17% of patients (n =7).

**Conclusions:**

The majority of nasal tubes are positioned in the middle nasal meatus. This result can be part of the answer to the question of the causal relationship between position of the breathing tube and the onset of maxillary sinusitis. The indications for prolonged nasotracheal intubation instead of orotracheal intubation or early tracheostomy should be considered carefully.

## Background

Despite the undisputed progress in modern intubation techniques and the options available for fixing common tube systems, there is an intrinsic issue of adverse consequences for transnasal intubation [1]. It is, therefore, no surprise that preference is frequently given to transoral procedures, submental intubation, or, in the case of a tracheotomized patient, to endotracheal intubation via the tracheostoma. However, it is accepted that a transnasal procedure cannot be avoided in many indications, particularly those involving maxillofacial surgery. In this context, numerous complications can occur, including soft-tissue and cartilaginous injuries as well as bone injuries in rare cases, delayed-onset damage, such as inflammation, ulcers, scars, and adhesions ranging as far as synechiae, and also drainage obstructions and paranasal sinusitis in long-term intubated patients [2,3].

However, if nasal intubation has to take precedence over anesthesia due to the specific problems associated with certain clinical scenarios, the disadvantages for the patient, who must be informed of them, are always a concern. Because the path of the tube in the nose, to name just one of the factors, entails very specific risks, it is useful to understand that path [4].

A survey of colleagues from different surgical specialties showed that the prevailing majority opinion is to place the tube in the inferior nasal meatus, but this does not, for example, adequately answer the question of the causal relationship between the position of the breathing tube and the onset of maxillary sinusitis. Besides addressing the surgery-specific issues, it seemed obvious also to consider the question of positioning the endonasal tube in patients who, for example, were already undergoing X-ray examination of all or part of the skull while under nasotracheal intubation anesthesia during dysgnathia surgery or jaw fracture treatment. This is particularly important in patients undergoing operations lasting for several hours or facing long-term ventilation. However, the literature clearly shows that ventilator-associated pneumonia (VAP) and nosocomial sinusitis in ICUs entail considerable dangers for the health and even the life of the patient [5-8]. A connection between these conditions and the tube seems reasonable; therefore, it is desirable to establish or rule out at an early stage whether the tube has directly or indirectly displaced the maxillary sinus ostium by moving the nasal soft tissues out of position.

## Methods

To obtain midface anatomical structures that were as intact as possible, patients who had been treated for isolated mandibular fractures were studied. In these patients, the endotracheal tube was inserted endonasally during intubation anesthesia to enable the surgeon to monitor occlusion after surgical treatment. The study protocol was approved by the Ethics Committee of the Hamburg Board of Physicians (permission number WF-013/13).

By means of the intraoperatively generated three-dimensional C-arm data sets (a standard procedure after the treatment of fracture patients), it was possible to determine the position of the nasotracheal tube during intubation anesthesia without exposing the patient to any additional radiation. The mobile C-arm Arcadis Orbic 3D (Siemens Medical Solutions, Erlangen, Germany) features an isocentric design and 190° orbital movement. The basic system consists of an isocentric C-arm with an integrated X-ray tube, opposite which is a 9-inch image intensifier. In our study, 50 to 100 two-dimensional images were taken between 30 and 60 s at a resolution of 1,024 × 1,024 pixels (1 K2). During scanning, a three-dimensional image data set (a cube approximately 12 cm^3^ in volume, or 2,563 voxels) was simultaneously calculated and displayed on the monitor in real time. During imaging, the correct positioning of the reconstructed data was visible, and the three-dimensional image data were immediately available after the scan had completed. These three-dimensional data were then visualized in multi-plane reconstructions (MPRs). In MPR mode, two-dimensional images of arbitrary orientation (axial, sagittal, coronal, oblique, double-oblique, and curvilinear) were calculated from the isotropic three-dimensional volume.

**Figure 1:**
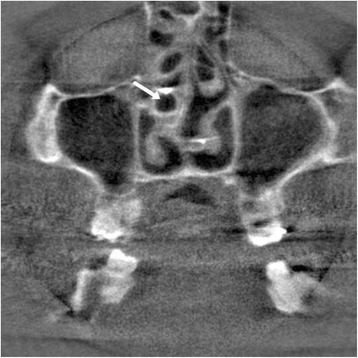
**Intraoperative multi**-**plane reconstruction showing the position of the tube in middle nasal meatus** **(arrow).**

Fifty data sets from patients who had undergone endonasal intubation were analyzed for tube positioning. In forty-two data sets it was possible to analyze the path of the tube in the nose. In eight data sets the tube was not to see. The analysis (30 minutes after intubation) involved 10 female patients (mean age: 39.7 years) and 32 male patients (mean age: 30.2 years). Tube positioning was determined at the maxillary sinus ostium in the intraoperatively generated three-dimensional X-ray data sets (see Figure 1). Different positions were distinguished: 1) tube positioned in the inferior nasal meatus (i); 2) tube positioned in the middle nasal meatus (m); and 3) tube positioned at the junction between the inferior and the middle nasal meatuses (mi). Moreover, the type of tube, the tube size, and the presence of maxillary sinusitis were established. All statistical analyses were performed with the statistical package R version 2.14.1. (R Foundation for Statistical Computing, Vienna, Austria, 2011; URL http://www.r-project.org).

## Results

**Figure 2:**
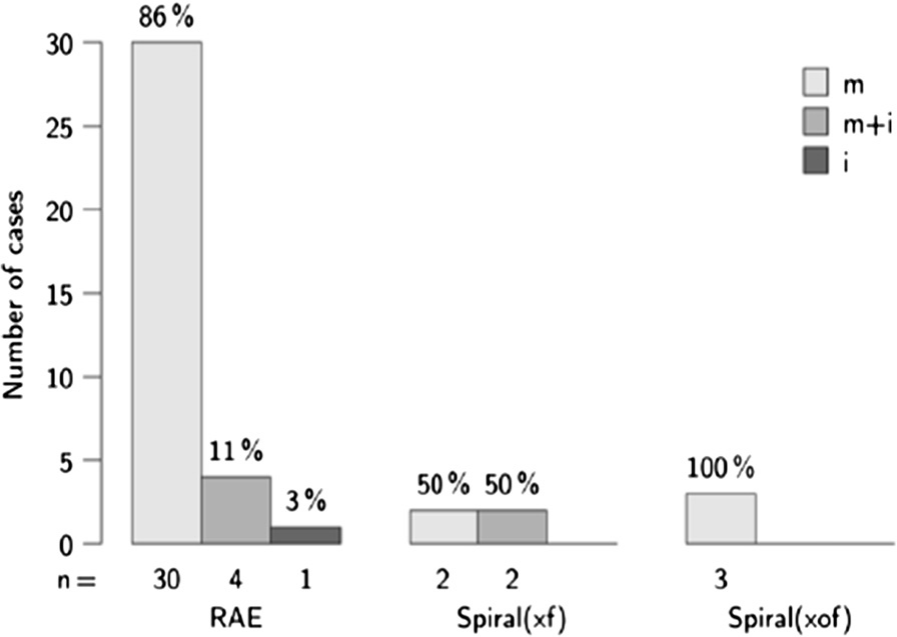
**Distribution of tube positioning** **(m:** **middle nasal meatus,** **m +** **i:** **middle and inferior nasal meatus,** **i:** **inferior nasal meatus).**

The test involved 42 of 50 subjects, in whom the tube was positioned in the middle nasal meatus in 35 (83.3%) patients and not in the middle nasal meatus in 7 (16.7%) patients (see Figure 2). The difference in comparison with an equal distribution was significant, with a *P*-value of <0.001. The test value was 83.3; the 95% confidence interval of the test value was 68.6 to 93.0%.

The RAE tube (Mallinckrodt, Glens Falls, NY, USA) was used in 83.3% of cases (n =35), the spiral (xf) in 9.5% of cases (n =4), and the spiral (xof) in 7.1% of cases (n =3). The difference in tube-type distribution was not significant. With a test size of χ^2^ = 5.1 and 4 degrees of freedom, the *P*-value was 0.281.

**Figure 3:**
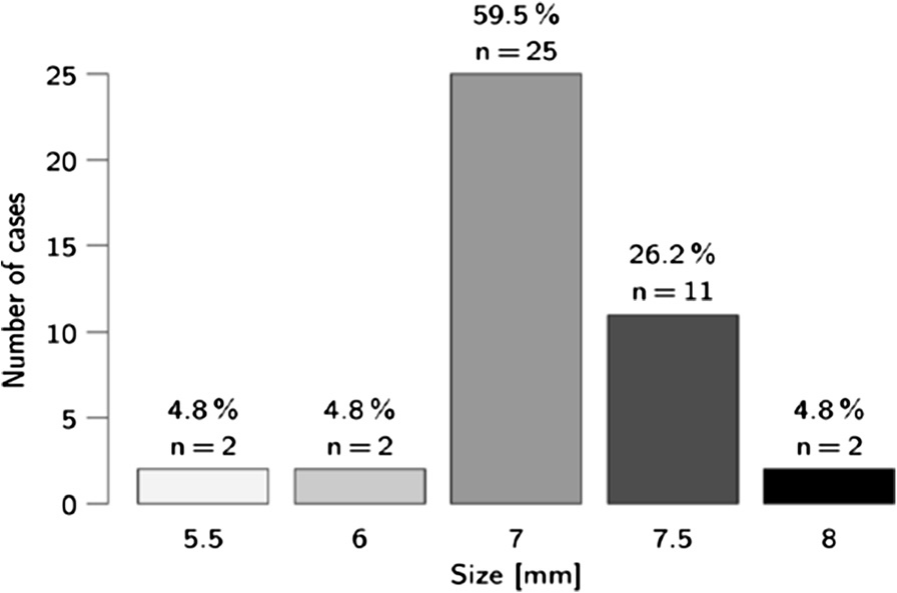
**Distribution of tube sizes.**

In 40.5% (n =17) of patients, the tube was positioned in the left nasal meatus, and in 59.5% of patients (n =25) it was positioned in the right nasal meatus. Figure 3 shows the tube size distribution. Maxillary sinusitis was diagnosed as a chance finding in 17% of patients (n =7).

## Discussion

With the development of low-pressure cuffs, prolonged nasotracheal intubation is a frequent occurrence. Laryngeal and tracheal complications from prolonged intubation are well recognized. Reports of maxillary sinusitis following nasotracheal intubation in this setting are appearing with increasing frequency [2,5-8]. We supposed the position of the tube to be in the inferior meatus but it was indeed mainly positioned in the middle nasal meatus. The pathogenesis of maxillary sinusitis is believed to be due to the development of edema in the nasal mucosa arising from irritation by the tube in the nasal cavity. The normal flora of the sinuses become pathogenic when trapped in a closed space. The fact that pathogenic microorganisms are found in fluid aspirated from the maxillary sinuses much more frequently in nasally-intubated patients than in orally-intubated patients may indicate that the nasal tube not only causes maxillary sinus drainage obstruction but can also encourage bacterial infection in the effusion, resulting in inflammation of the maxillary sinus wall [6,7]. The infection is often caused by Gram-negative bacteria [9,10]. An incidence of 0.3% in short-term intubations (less than 5 days) and 40.4% in long-term intubations (more than 5 days) has been reported. In neurosurgical patients treated with nasotracheal intubation (NTI), sinusitis is found in 52 to 100% of patients [8]. Our cases of identified maxillary sinusitis were most likely not induced by the tube because the period for which the nasal tube was in position was unlikely to have been long enough to cause sinusitis. In relation to the cases of paranasal inflammation described in the literature, this raises the question of whether sinusitis may have already been present beforehand, or have been induced by the tube [5-8,11].

It is likely that the circumstances of the ICU might also be of major importance in the development of maxillary sinusitis in ventilated patients; this includes a prolonged time in the supine position and immobility, as well as treatment with mechanical ventilation and sedation over a long period. Even in healthy human subjects, the functional size of the maxillary ostium is significantly reduced in a semi-reclined or lying position due to diminished venous blood flow from the head and neck region. Additionally, in ICU patients, ventilatory support with positive expiratory and end-expiratory pressure induces an increase in central venous pressure, thus intensifying these effects. Moreover, in this situation, patients are unable to use normal physiological cleansing mechanisms, such as coughing and sneezing, which enhance flow rates through the pharynx and nose [5-7]. Therefore, mechanical obstruction of the sinus ostia by the nasal tube and the concomitant conditions of intensive care lead to a dynamic process characterized by edema, diminished mucociliary cleansing of the sinus cavity, proliferating sinus flora, and inflammation [5-7].

In adults, the indications for prolonged NTI instead of orotracheal intubation and early tracheostomy should be considered carefully. If fever or sepsis develops in patients treated with NTI, investigations for sinusitis should be included [12,13]. When sinusitis has developed, the tube should be removed and the patient tracheostomized. Otherwise, surgical drainage should be performed.

## Conclusions

Eighty percent of nasal tubes are positioned in the middle nasal meatus and are, therefore, associated with the theoretical risk of causing septic complications due to mechanical displacement of the maxillary sinus ostium, particularly in long-term ventilated patients. Nasotracheal intubation should be regarded as an additional risk for the development of sinusitis and other adverse effects, such as synechiae, and so on [13]. The indications for prolonged nasotracheal intubation, instead of orotracheal intubation or early tracheostomy, should be considered carefully in long-term ventilated patients, as should changing the tube from the right to left nostril, carrying out decongestant procedures, and aspirating the maxillary sinuses specifically or via prophylactic maxillary sinus drainage. However, it is clear that, when appropriately indicated, nasal intubation may be the optimum procedure.

## Abbreviations

ICU: Intensive care unit
i: Inferior nasal meatus
m: Middle nasal meatus
mi: Junction betwee the inferior and the middle nasal meatuses
MRP: Multi-plane reconstruction
VAP: Ventilator-associated pneumonia
xf: fiberoptic intubation
xof: without fiberoptic intubation
